# The Use of AngioVac in a Patient With Severe Infective Endocarditis Where Open Heart Surgery Is Contraindicated: A Case Report

**DOI:** 10.7759/cureus.39639

**Published:** 2023-05-29

**Authors:** Duo Xiao, Raeann Dalton, Adam Fineman, Michael Benz, Antonios Tsompanidis

**Affiliations:** 1 Anesthesiology, Rowan-Virtua School of Osteopathic Medicine, Stratford, USA; 2 Urology, Rowan-Virtua School of Osteopathic Medicine, Stratford, USA; 3 Cardiology, CarePoint Health-Christ Hospital, Jersey City, USA; 4 Structural Heart Program, Jersey City Medical Center, Jersey City, USA; 5 Cardiology, Rutgers University New Jersey Medical School, Newark, USA; 6 Cardiology and Catheterization Laboratory, CarePoint Health-Christ Hospital, Jersey City, USA; 7 Graduate Medical Education, CarePoint Health-Christ Hospital, Jersey City, USA; 8 Graduate Medical Education, CarePoint Health-Bayonne Medical Center, Bayonne, USA

**Keywords:** debulk, tricuspid vegetation, streptococcus mitis, angiovac system, tricuspid valve endocarditis

## Abstract

Infectious endocarditis of the tricuspid valve (TV) is a common complication of intravenous (IV) drug use. Endocarditis caused by viridans streptococci can lead to heart valve vegetations, which may be life-threatening due to the potential for embolism and obstruction. The management of large valvular vegetations is often difficult due to the risks involved with open heart surgery, especially in patients with comorbid conditions. The AngioVac device (AngioDynamics Inc., Latham, NY) has been shown in rare cases to be effective at debulking vegetations without the need for invasive surgery. We present a 45-year-old male with a history of intravenous heroin use disorder, hepatitis C, spinal abscesses, and chronic anemia who experienced worsening shortness of breath, generalized weakness, bilateral lower extremity edema, dysuria with dark urine, and blood on toilet paper. Workup revealed a 4.39 × 4.35 cm tricuspid valve vegetation, severe tricuspid regurgitation (TR), acute renal failure, acute on chronic anemia, and thrombocytopenia from sepsis-induced disseminated intravascular coagulation (DIC). AngioVac was used to aspirate the vegetation and effectively reduced the size to 3.75 × 2.31 cm. Follow-up blood cultures revealed no growth after five days. This is the largest documented tricuspid valve vegetation with a successful implementation of the AngioVac to date. This therapy, in conjunction with intravenous antibiotics and hemodialysis, successfully sterilized the vegetation, prevented worsening presentation, and averted life-threatening complications, although severe tricuspid regurgitation persisted. Based on the findings of this case, the AngioVac device is a safe and effective treatment option for tricuspid valve endocarditis patients with large vegetation and severe comorbidities, which contraindicate open heart surgery.

## Introduction

Infective endocarditis (IE) is a microbial infection affecting the inner layer of the heart, predominantly the heart valves. Etiologies of infection commonly stem from congenital or acquired valve disease, indwelling catheters, dental procedures, intravenous (IV) drug use disorder, prosthetic valves, and implanted cardiac devices [1,2]. Approximately 80%-90% of the organisms causing IE are *Staphylococcus*, *Streptococcus*, and *Enterococcus *spp., with *Staphylococcus aureus* being the most isolated organism associated with IE [3]. IE commonly leads to heart valve vegetations, causing a myriad of symptoms including fever, shortness of breath, chest pain, swelling in the legs, painful nodules on the fingertips (Osler nodes), painless lesions on the hands and feet (Janeway lesions), small hemorrhages in the nail bed and eyes, and flu-like symptoms [4]. Severe IE can also lead to life-threatening complications such as heart failure, pulmonary embolism, and stroke if not adequately addressed [4,5].^ ^This case presents a patient with a history of IV drug abuse and a relatively large tricuspid valve (TV) vegetation comorbid with thrombocytopenia, anemia, and acute renal failure. Due to the risks involved with open heart surgery, a minimally invasive procedure involving the AngioVac device (AngioDynamics Inc., Latham, NY) was implemented to debulk the vegetation in an effort to decrease the likelihood of severe, life-threatening complications. This article was previously presented as a poster at Rowan-Virtua School of Osteopathic Medicine (SOM) Research Day on May 4, 2023.

## Case presentation

A 45-year-old Hispanic male with a history of bipolar disorder, heroin use disorder, spinal abscesses, hepatitis C, and chronic anemia presented to the ED with worsening shortness of breath, generalized weakness, bilateral lower extremity edema, dysuria with dark urine, and blood on toilet paper (but not in stool). On review of systems, the patient denied any fever, headaches, chest pain, cough, headaches, skin changes, and diarrhea. The patient did report recent nausea and vomiting with no blood. Social history was significant for daily IV heroin use; the patient did not feel comfortable disclosing when he first began using heroin. The patient denied any alcohol or tobacco usage. Family history is unknown.

A transthoracic echocardiogram (TTE) was done and showed a TV vegetation measuring 4.39 × 4.35 cm with severe tricuspid regurgitation (TR). Blood cultures on admission and one day following admission both yielded gram-positive cocci; urine cultures showed no growth. Further evaluation showed the presence of *Streptococcus mitis*, a subspecies of viridans group streptococci. The patient was empirically started on IV Zosyn and Vancomycin.

Due to the large size, the TV vegetation was unlikely to be eradicated with medical therapy alone. Further evaluation of the patient demonstrated acute renal failure, acute on chronic anemia, and thrombocytopenia from sepsis-induced disseminated intravascular coagulation (DIC). Due to the aforementioned circumstances, the patient was not a candidate for open heart surgery for the removal of the TV vegetation. Minimally invasive utilization of the AngioVac device was justified, and the use of the AngioVac was reviewed with the patient and family.

**Figure 1:**
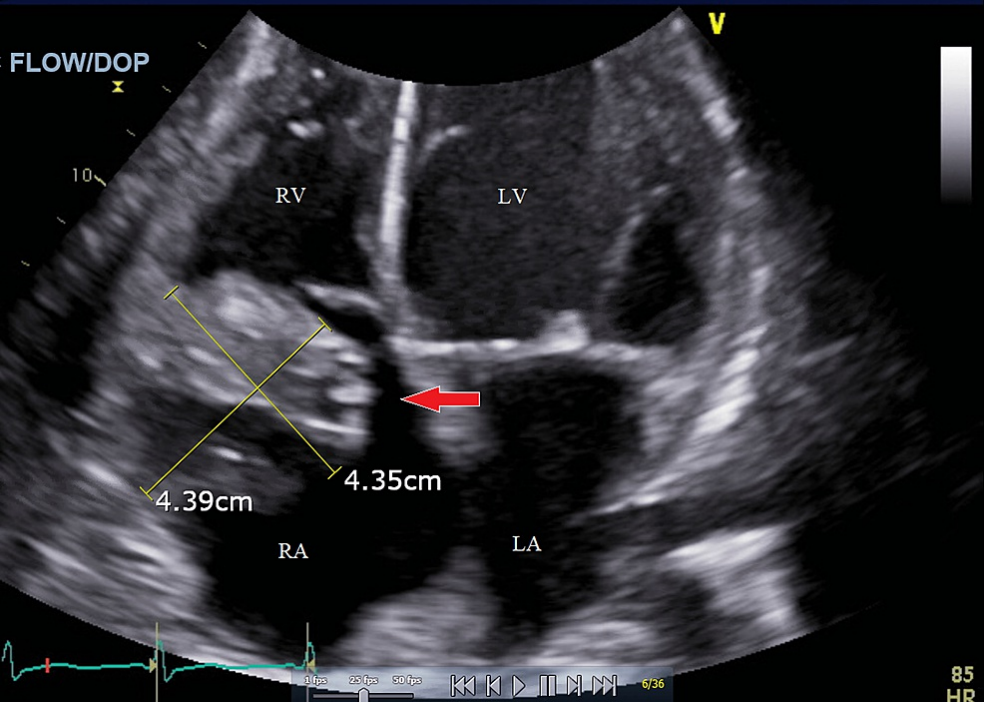
Transthoracic echocardiogram via four-chamber view with a red arrow pointing to the vegetation before the AngioVac procedure. RA, right atrium; RV, right ventricle; LA, left atrium; LV, left ventricle

**Figure 2:**
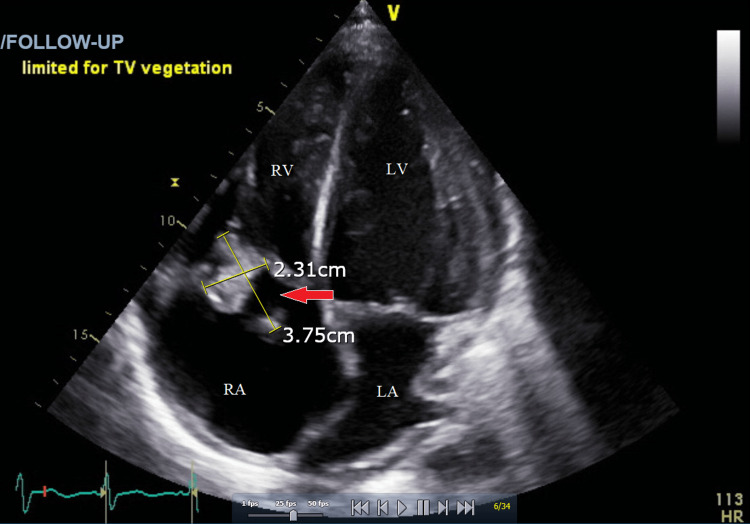
Transthoracic echocardiogram via four-chamber view with a red arrow pointing to the vegetation after the AngioVac procedure. RA, right atrium; RV, right ventricle; LA, left atrium; LV, left ventricle; TV, tricuspid valve

Following aspiration, the TV vegetation was effectively reduced from 4.39 × 4.35 cm to 3.75 × 2.31 cm. One day following the procedure, three separate blood cultures were obtained, ultimately leading to no growth after five days; the remnant TV vegetation was deemed sterile. The patient showed resolution of initial presenting symptoms; however, the following TTE after AngioVac use still showed severe tricuspid regurgitation due to the lingering 3.75 × 2.31 cm vegetation. Seven days following the procedure, the patient left the hospital against medical advice (AMA). The figures below show the echocardiogram of the vegetation before (Figure 1) and after (Figure 2) the procedure that ultimately leads to the stabilization of the patient presenting symptoms.

## Discussion

AngioVac is a percutaneous vacuum-based device able to aspirate materials from the intravascular system [6,7]. First approved for use in 2014, AngioVac was commonly used to aspirate iliocaval, pulmonary, upper extremity, and right heart chamber thrombi [7]. The current literature regarding the use of AngioVac in TV endocarditis is limited. However, recent case reports, as well as a single case series, have demonstrated its efficacy in treating patients with bacterial TV vegetations with multiple comorbidities [6-10]. The majority of published cases using the AngioVac were successful in debulking the vegetation and improving acute patient symptoms, but at this time, only a single case in the literature was successful in entirely removing a TV vegetation [7]. With this in mind, the patient was deemed an adequate candidate for the procedure due to his contraindications for open heart surgery and the high likelihood of improvement with AngioVac [11].

This case report demonstrates the viability of AngioVac in a singular patient with TV endocarditis where open heart surgery was contraindicated. To our knowledge, it is the largest TV vegetation in the current literature to be debulked using the AngioVac device [6-10]. Although severe tricuspid regurgitation still remained due to the remnant vegetation, the procedure was successful in completely sterilizing the patient from the inciting microorganism. A major reason why the procedure was halted preemptively was due to the fear of further complications, namely, the fear of severely damaging the tricuspid valve. Nevertheless, reducing the size of the vegetation allowed for adequate antibiotic penetration. The procedure persisted for over two hours until the cessation of the procedure was agreed upon. Collaboratory with IV antibiotics and hemodialysis, debulking the thrombus was necessary to resolve the present symptoms, prevent the worsening presentation of the patient, and avert future life-threatening complications.

## Conclusions

AngioVac proved to be an effective alternative to treating a severe case of tricuspid valve endocarditis where cardiothoracic surgery was contraindicated due to comorbid diseases such as thrombocytopenia, anemia, and acute renal failure. Debulking the vegetation with AngioVac led to the improvement of initial symptoms and the complete sterilization of the offending microbe.
